# The Combination of *Pterocarpus marsupium* Bark Extract, *Pinus strobus* Bark Extract, and Ascorbyl Tetraisopalmitate Inhibits Melanogenesis via Nicotinamide Nucleotide Transhydrogenase Activation

**DOI:** 10.1111/jocd.70606

**Published:** 2025-12-17

**Authors:** Kejie Peng, Jian Mu, Yumeng Li, Zhangtie Wang, Yansong Peng, Tian Zhao, Yixuan Wang, Jing Wang, Baiyi Lu

**Affiliations:** ^1^ College of Biosystems Engineering and Food Science, Key Laboratory for Quality Evaluation and Health Benefit of Agro‐Products of Ministry of Agriculture and Rural Affairs, Key Laboratory for Quality and Safety Risk Assessment of Agro‐Products Storage and Preservation of Ministry of Agriculture and Rural Affairs Zhejiang University Hangzhou China; ^2^ ZJU‐Hangzhou Global Scientific and Technological Innovation Center Hangzhou China; ^3^ Proya Cosmetics Co. Ltd Hangzhou China

**Keywords:** ascorbyl tetraisopalmitate, melanogenesis, nicotinamide nucleotide transhydrogenase, *Pinus strobus* bark extract, *Pterocarpus marsupium* bark extract

## Abstract

**Background:**

Excessive ultraviolet exposure triggers skin pigment deposition, potentially leading to conditions such as melasma, freckles, and post‐inflammatory hyperpigmentation. The skin of individuals with these dermatological conditions exhibits reduced levels of nicotinamide nucleotide transhydrogenase (NNT). While many seek skin‐whitening products to address pigmentation, existing options often fail to achieve optimal efficacy. To accomplish a synergistic whitening effect, we developed PPV, a composite formulation incorporating three depigmenting agents: *Pterocarpus marsupium* bark extract (PMBE), *Pinus strobus* bark extract (PSBE), and ascorbyl tetraisopalmitate (VC‐IP).

**Aims:**

This study aimed to investigate the effects of PPV on skin whitening and elucidate its mechanisms.

**Methods:**

After administering PPV to B16F10 melanoma cells, we measured melanin content and gene expression of *TYR*, *TRP‐1*, and *TRP‐2* using a microplate reader and qPCR. Western blotting was used to assess p‐CREB, CREB, MITF, and NNT expression. The Jin's formula was used to evaluate the combined effect of the three components of PPV on *NNT* at the genetic level. Oxidative stress levels were evaluated with ROS, GSH/GSSG, and NADP^+^/NADPH assay kits, along with confocal microscopy. Transmission electron microscopy and ELISA were employed to quantify the eumelanin/pheomelanin ratio and melanosome maturation. Finally, immunodetection was performed to assess epidermal basal pigmentation, tyrosinase, and NNT expression in a human ex vivo skin model.

**Results:**

PPV significantly influenced melanin production in melanocytes and human ex vivo skin. This effect was due to the inhibitory effect of PPV on the CREB‐MITF signaling pathway and the synergistic activation effect of PMBE, PSBE, and VC‐IP on the *NNT* gene expression in B16F10 cells. Activation of NNT led to a reduction in ROS levels and the NADP^+^/NADPH ratio, ultimately inhibiting melanogenesis, tyrosinase activity, and melanosome maturation in B16F10 cells. Furthermore, PPV treatment reduced pigmentation and increased NNT expression in the aged ex vivo skin model.

**Conclusion:**

PPV, synthesized from PMBE, PSBE, and VC‐IP, exhibited an anti‐melanogenic effect. This effect was attributed to the inhibition of CREB‐MITF‐mediated melanogenesis and the regulation of oxidative stress by NNT. The findings suggest PPV as a potential therapeutic agent for skin pigmentation disorders.

**Abbreviations**
2,3BD: 2,3‐butanedione
PMBE:
*Pterocarpus marsupium* bark extract
PPV: a mixture of *Pterocarpus marsupium* bark extract, *Pinus strobus* bark extract, and ascorbyl tetraisopalmitate
PSBE:
*Pinus strobus* bark extract
TRP‐1: tyrosinase‐related protein 1
TRP‐2: tyrosinase‐related protein 2
TYR: tyrosinase
VC‐IP: ascorbyl tetraisopalmitate

## 1 Introduction

Upon exposure to ultraviolet B (UVB) radiation, melanin is synthesized in melanocytes to protect the skin [1]. Melanin synthesis involves the transformation of tyrosine by enzymes such as tyrosinase (TYR), tyrosinase‐related proteins 1 (TRP‐1), and tyrosinase‐related proteins 2 (TRP‐2), which are regulated by the microphthalmia‐associated transcription factor (MITF) [2]. The melanogenic pathway initiates with TYR‐catalyzed hydroxylation of tyrosine to l‐dihydroxyphenylalanine (l‐DOPA), followed by its oxidation to dopaquinone (DQ). This unstable quinone derivative undergoes spontaneous cyclization to form dopachrome [3], which is subsequently stabilized through TRP‐2‐mediated conversion into 5,6‐dihydroxyindole‐2‐carboxylic acid (DHICA) [4]. The final oxidative polymerization of DHICA by TRP‐1 yields eumelanin polymers [5], though this pathway can be redirected toward pheomelanin synthesis when cysteine or reduced glutathione (GSH) is present [6]. Concurrent with pigment synthesis, melanosome maturation progresses through four distinct morphological stages (I–IV). Early stage melanosomes (stage I/II) establish their amyloid matrix through proteolytic processing of premelanosome protein (PMEL) [7]. As melanin accumulates, melanosomes darken and enlarge, progressing to stages III and IV, accompanied by a dynamic pH shift from 5.0 to 6.8 within the maturing organelle [8].

Emerging evidence revealed alternative pigmentation regulators beyond canonical UVB and MITF signaling, with mitochondrial nicotinamide nucleotide transhydrogenase (NNT) demonstrating redox‐dependent modulation of melanogenesis. This mitochondrial inner membrane protein transfers electrons from NADH to NADP^+^ to produce NADPH [9], establishing an elevated NADPH‐dependent GSH/GSSG ratio that critically influences pheomelanin and eumelanin production [10]. Mechanistically, silencing the expression of NNT can increase TYR, TRP‐1, and TRP‐2 protein levels and promote melanosome maturation through redox reactions, operating independently of both UVB exposure and MITF regulation [11].

Contemporary depigmentation strategies increasingly employ multi‐target formulations to address melanin deposition. The novel PPV compound combined *Pterocarpus marsupium* bark extract (PMBE), *Pinus strobus* bark extract (PSBE), and ascorbyl tetraisopalmitate (VC‐IP). This synergistically targets distinct pigmentation pathways: (1) PMBE downregulated cAMP‐response element binding protein (CREB)–MITF signaling to suppress TYR/TRP‐1/TRP‐2 expression [12]; (2) PSBE, primarily composed of procyanidins, induced melanocyte apoptosis via mitochondrial‐associated pathways [13], while its phenolic compounds provided antioxidant protection [14]; and (3) the lipophilicity of VC‐IP enhanced the bioavailability of vitamin C, effectively counteracting UVB‐induced pigmentation by inhibiting tyrosinase and promoting dermal restructuring [15, 16]. Notably, while individual components demonstrate efficacy against pigmentation, their combined effects on NNT remain unexplored, particularly regarding redox modulation and melanosome maturation.

In this study, we aimed to evaluate the efficacy of the PPV compound in inhibiting melanogenesis, as well as to elucidate its underlying mechanisms. Our findings indicated that PPV reduced melanin production by downregulating the CREB‐MITF pathway and upregulating NNT expression. Further analysis revealed that PPV also mitigated intracellular oxidative stress and inhibited melanin maturation, thereby reducing melanin deposition. Importantly, these effects were mediated through NNT, highlighting its pivotal role in the PPV‐driven suppression of melanogenesis. Finally, we verified the whitening effect of PPV in an ex vivo skin model.

## 2 Materials and Methods

### 2.1 Chemicals and Reagents

PPV was prepared as a liquid formulation prior to application. The PPV solution contained 1 mg/mL of PPV, which included the following components: 0.1358 mg/mL of PMBE (purity > 70%, Sabinsa, USA), 0.247 mg/mL of PSBE (purity 2%–3.5%, Lucas Meyer, Switzerland), and 0.6172 mg/mL of VC‐IP (purity ≥ 99%, BERI PHARMA, China). 2,3BD was obtained from Aladdin (Shanghai, China).

### 2.2 Cell Cultures

B16F10 cells were obtained from Shanghai EK‐Bioscience Biotechnology Co. Ltd. (Shanghai, China). Cultured in RPMI 1640 medium supplemented with 10% fetal bovine serum (TIANHANG, China) and 1% penicillin/streptomycin (Gibco, USA), cells were maintained at 37°C in 5% CO_2_.

### 2.3 Cell Viability

Cell viability was assessed using the Cell Counting Kit‐8 (CCK‐8, Yeasen, China). B16F10 cells treated with various concentrations of therapeutic agents for 48 h were incubated with CCK‐8 solution for 1 h at 37°C. Absorbance at 450 nm was measured using a microplate reader (Biotek, USA).

### 2.4 Inhibition Rate of Melanin Content

Melanin content was assessed as previously described [17]. B16F10 cells were treated with therapeutic agents for 48 h. After cell collection and centrifugation, 1% Triton X‐100 (Solarbio, China) in phosphate‐buffered saline (PBS, Solarbio, China) was added, followed by a freeze–thaw cycle. The supernatant and precipitate were separated. 1 M NaOH was added to the precipitate and heated at 80°C for 10 min. Absorbance was measured at 405 nm.

### 2.5 Inhibition Rate of Tyrosinase Activity

50 μL of supernatant, after melanin removal, was mixed with 150 μL of 1 mg/mL l‐DOPA (Solarbio, China). After 30 min of incubation at 37°C, absorbance was measured at 475 nm.

### 2.6 qPCR

Total RNA was extracted from B16F10 cells and assessed for concentration and purity. Reverse transcription was performed using the SPARKscript II RT Plus Kit (Sparkjade, China). qPCR analysis was conducted using the 2×SYBR Green qPCR Mix (Sparkjade, China) with primers listed in Table 1. Results were normalized to β‐actin expression.

**TABLE 1:** qPCR primer sequences.

Gene	Forward primer	Reverse primer
*NNT*	CAGCTCTGATTCCAGGTGGTT	ATACTCTGGAGGGTCCGTGG
*TYR*	TCACCATGCTTTTGTGGACAGTATT	TCTGTTATGGCCGATAGGTGC
*TRP‐1*	CAGGCAATACAACATGGTGCC	ATACAGTAAACTCCTGACCTGGC
*TRP‐2*	ACCCATTGGTCACAACCGAA	AACTGGAGCTTCTTCCTCTGAC
*β‐actin*	GGCTGTATTCCCCTCCATCG	CCAGTTGGTAACAATGCCATGT

### 2.7 Synergistic Interaction Analysis

The synergistic efficacy of PMBE, PSBE, and VC‐IP was evaluated by means of Jin's formula, with *Q* < 0.55, 0.55–0.85, 0.85–1.15, and ≥ 1.15 indicating significant antagonism, antagonism, additivity, and synergism, respectively [18]. Jin's formula is as follows:

$$Q=\frac{E\left(a+b+c\right)}{\left(\mathrm{Ea}+\mathrm{Eb}+\mathrm{Ec}-\mathrm{Ea}\times \mathrm{Eb}\times \mathrm{Ec}\right)}$$

where *E*(*a* + *b* + *c*), *Ea*, *Eb*, and *Ec* are the average effects of the combination treatment, PMBE alone, PSBE alone, and VC‐IP alone, respectively.

### 2.8 Western Blotting

The method was adapted from a previous study [19]. B16F10 cell proteins were extracted using RIPA lysis buffer (Beyotime, China). Protein concentrations in each group were determined using a BCA protein assay kit (Beyotime, China), and absorbance was measured at 562 nm. Proteins were separated by 10% Tris‐Glycine sodium dodecyl sulfate (SDS)‐polyacrylamide gel electrophoresis (PAGE), followed by transfer to polyvinylidene fluoride (PVDF) membranes (Millipore, USA). The membranes were blocked with 5% skim milk (i‐presci Scientific, China) in Tris‐buffered saline with Tween‐20 for 2 h and then incubated with primary antibodies at 4°C for 12–16 h. Membranes were incubated with secondary antibodies at room temperature for 2 h. Immunoreactive bands were visualized using ECL chemiluminescence substrate. Protein bands were quantified using Chemi Analysis software. The following antibodies were used: NNT (Proteintech, China), MITF (Santa Cruz, USA), p‐CREB (Affinity, China), CREB (Affinity, China), GAPDH (Servicebio, China), β‐actin (Servicebio, China), and anti‐rabbit (Affinity, China). Band intensities were semi‐quantified using ImageJ and normalized to the β‐actin or GAPDH band.

### 2.9 Measurement of Oxidative Stress Level

The intracellular ROS content, NADP^+^/NADPH ratio, and GSH/GSSG ratio were measured using appropriate assay kits purchased from Beyotime (Shanghai, China).

### 2.10 Mitochondrial Oxidative Stress Level Measurement

Mito‐Tracker Red CMXRos (Beyotime, China) was used to measure mitochondrial oxidative stress level. Cells were incubated with Mito‐Tracker Red CMXRos (200 nM) at 37°C for 30 min, then stained with 4′,6‐diamidino‐2‐phenylindole (DAPI). Images were acquired using a confocal microscope (LSM 880, ZEISS), processed with ZEN2010 software, and quantified using ImageJ.

### 2.11 Eumelanin/Pheomelanin Ratio Assay

The eumelanin and pheomelanin contents were determined using appropriate ELISA kits provided by Shanghai Enzyme‐linked Biotechnology Co. Ltd. The cell supernatant was collected for subsequent analysis according to the manufacturer's instructions.

### 2.12 Measurement of Intracellular pH

Cells were incubated with 10 μM 2′,7′‐bis‐(2‐carboxyethyl)‐5‐(and‐6)‐carboxyfluorescein, acetoxymethyl ester (BCECF AM, Beyotime, China) at 37°C for 30 min. Fluorescence intensity was then measured using a fluorescence spectrophotometer (Molecular Devices, USA) with excitation/emission wavelengths of 488 nm/535 nm.

### 2.13 Transmission Electron Microscope

Cells were fixed in 2.5% glutaraldehyde (Yuanye, China), dehydrated in a graded ethanol series for 15 min each, and infiltrated with a 1:1 ethanol‐embedding medium mixture for 1 h, followed by overnight infiltration with pure embedding medium. A final infiltration step with fresh embedding medium was performed. Samples were embedded at 80°C for 8 h, trimmed, and stained with uranyl acetate and lead citrate for electron microscopy. Thin sections were examined using an HT7820 transmission electron microscope (Hitachi, Japan).

### 2.14 Human Ex Vivo Skin Model

Human skin specimens were obtained from BIOCELL (Guangdong, China) with ethical consent (approval No. GDLL2024002 and No. GDLL2024003). Young ex vivo skin was obtained from the abdomen of a 30‐year‐old female volunteer. Aged ex vivo skin was obtained from the abdomen of a 42‐year‐old female volunteer. Following mechanical processing to remove subcutaneous adipose layers, the tissue samples were positioned in six‐well culture plates with epidermal surfaces oriented superiorly. Specimens were maintained in a controlled environment (37°C, 5% CO_2_ atmosphere) using standard cell culture protocols. Throughout the experimental period, the culture medium was refreshed daily. Pharmacological intervention commenced 24 h post‐culture initiation, with therapeutic agents being administered consecutively for 7 days.

### 2.15 Immunohistochemical Staining

Protocols from previous studies were applied to assess pigmentation levels and tyrosinase content [20]. Antigen retrieval was performed using boiling citrate buffer, followed by incubation with samples to inactivate endogenous peroxidases. Skin sections were then incubated with anti‐tyrosinase antibodies (Abcam, China). Masson‐Fontana staining was conducted following the guidelines of the Masson‐Fontana Melanin Stain Kit (Solarbio, China). Skin sections were observed under a BX53 microscope (Olympus, Japan).

### 2.16 Immunofluorescence Assay

The NNT expression was assessed according to previously established methods [21]. Tissue samples were arranged in a 6‐well plate with the epidermal side up. After treatment, the samples were fixed in 4% paraformaldehyde (Biosharp, China) for 24 h. Tissues were incubated with anti‐NNT antibodies (Abcam, China) overnight at 4°C. Secondary antibodies were introduced for 2 h at room temperature. Nuclei were counterstained with DAPI. Images of the immunostaining were captured using a BX43 fluorescence microscope (Olympus, Japan).

### 2.17 Statistical Analysis

All experimental data were expressed as mean ± standard deviation derived from a minimum of three independent biological replicates. Graphical representations were generated through GraphPad Prism 8 software with statistical evaluations implemented in SPSS Statistics 26.0. One‐way analysis of variance (ANOVA) followed by LSD's test or Dunnet's test was used for data comparison.

## 3 Results

### 3.1 The Cytotoxicity of PMBE, PSBE, VC‐IP, and PPV on B16F10 Melanoma Cells

**FIGURE 1:**
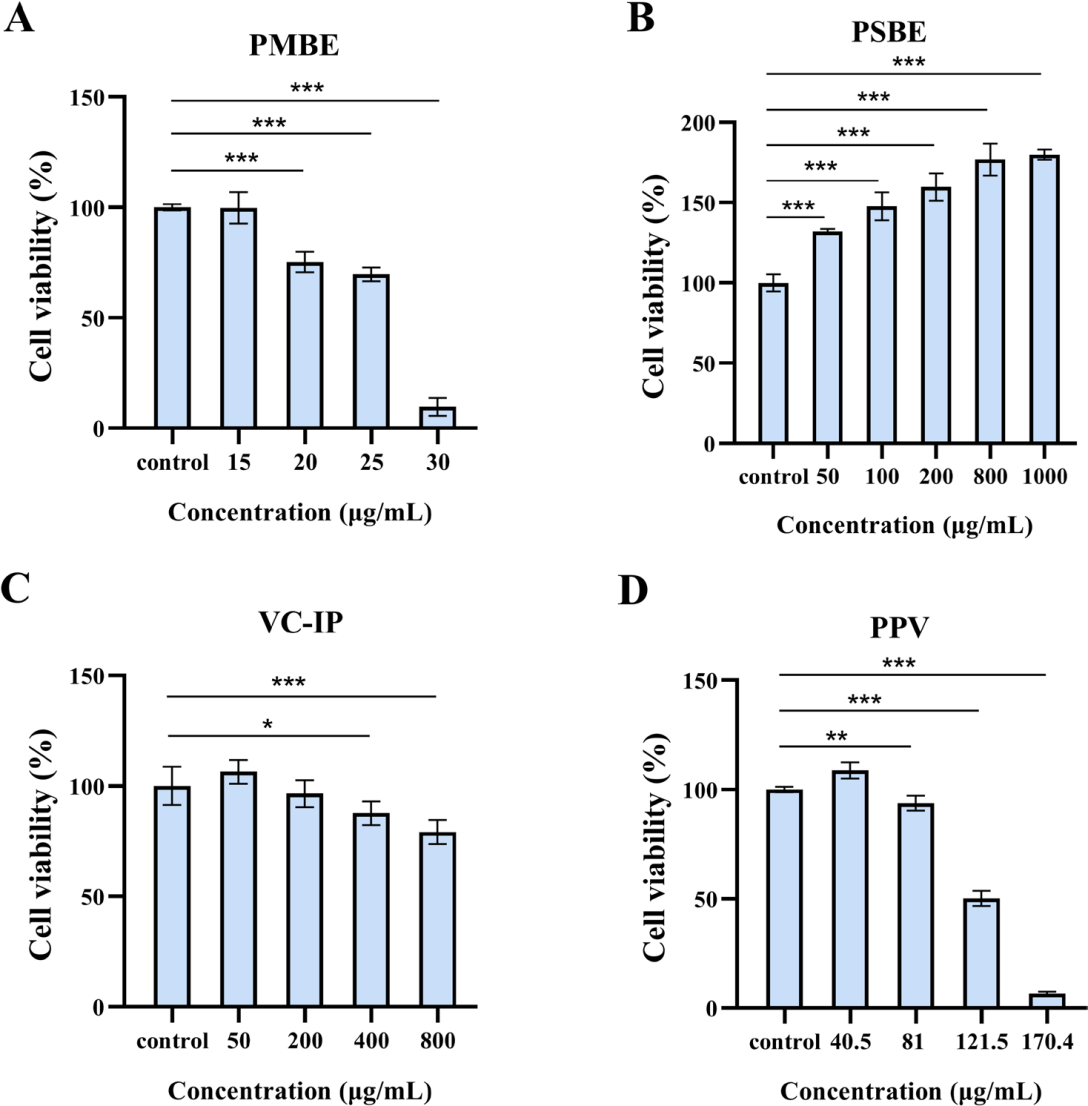
The cytotoxicity of PMBE, PSBE, VC‐IP and PPV on B16F10 melanoma cells. The viability of B16F10 cells after 48 h treated with (A) PMBE, (B) PSBE, (C) VC‐IP and (D) PPV was assessed using the CCK‐8 assay. Data were presented as the mean ± SD of at least three independent experiments. The statistical analysis was performed using one‐way ANOVA followed by LSD's post hoc test. *p < 0.05, **p < 0.01, and ***p < 0.001 versus control groups. PMBE, *Pterocarpus marsupium* bark extract; PSBE, *Pinus strobus* bark extract; VC‐IP, ascorbyl tetraisopalmitate; PPV, a combination of PMBE, PSBE, and VC‐IP.

The cytotoxic effects of varying concentrations of PMBE (Figure 1A), PSBE (Figure 1B), VC‐IP (Figure 1C), and the combined formulation PPV (Figure 1D) were systematically assessed using the CCK‐8 assay in B16F10 melanoma cells. PMBE demonstrated concentration‐dependent cytotoxicity, with no significant reduction in cell viability observed at concentrations ≤ 15 μg/mL. However, exposure to 20 μg/mL PMBE resulted in a marked decrease in cell survival to 75.20%. Notably, PSBE exhibited a proliferative effect at concentrations below 1000 μg/mL, showing no cytotoxic inhibition. VC‐IP maintained cellular viability above 95% at concentrations ≤ 200 μg/mL, indicating negligible cytotoxicity. The PPV formulation, containing cumulative concentrations of PMBE, PSBE, and VC‐IP, showed no significant cytotoxic effects at concentrations ≤ 40.5 μg/mL. Based on these dose–response relationships, subsequent experiments employed concentrations of 1, 10, and 25 μg/mL to ensure cellular viability.

### 3.2 Inhibition Effects of PPV on Melanogenesis and Tyrosinase Activity

**FIGURE 2:**
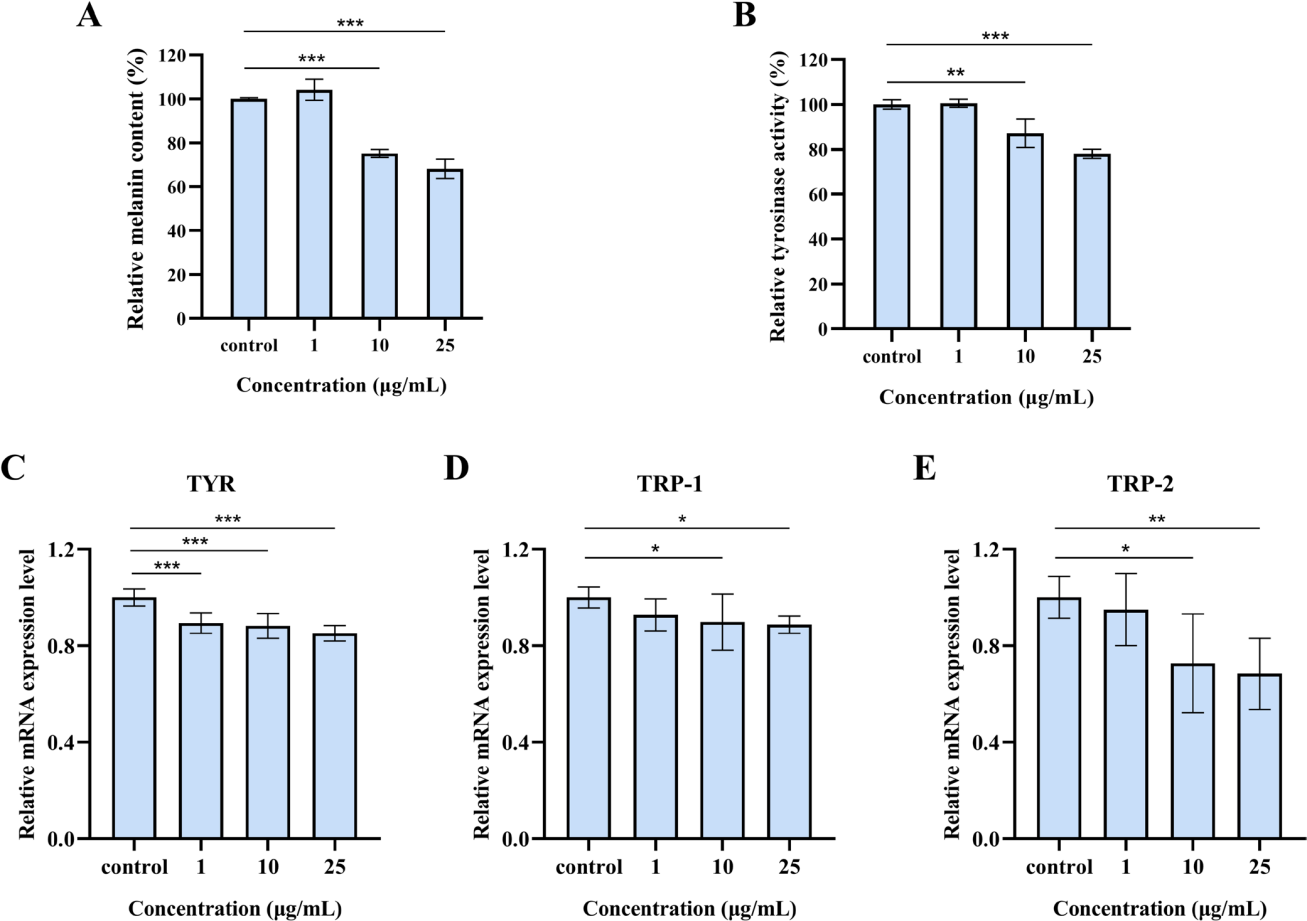
Effects of PPV on melanin content, tyrosinase activity, and pigmentation‐related genes. (A) The relative melanin content and (B) tyrosinase activity were measured in PPV‐treated B16F10 cells. The relative mRNA expression levels of (C) *TYR*, (D) *TRP‐1*, and (E) *TRP‐2* were assessed after 48 h of PPV treatment. Data were presented as the mean ± SD of at least three independent experiments. The statistical analysis was performed using one‐way ANOVA followed by LSD's post hoc test. *p < 0.05, **p < 0.01, and ***p < 0.001 versus control groups.

The effects on relative melanin content were evaluated at concentrations of 1, 10, and 25 μg/mL. Treatment with PPV led to a dose‐dependent reduction in melanin content, with the highest decrease observed to be 32% at 25 μg/mL (Figure 2A). Tyrosinase activity is essential for melanin production [22]. l‐DOPA oxidation assay was used to assess the effect of PPV on cellular tyrosinase activity. As shown in Figure 2B, tyrosinase activity exhibited parallel dose‐responsive inhibition, with peak suppression reaching 22% at the highest concentration tested.

Melanocytes possess unique enzymes, including TYR, TRP‐1, and TRP‐2, that are crucial for melanin production [22]. We quantified mRNA expression of core melanogenic regulators (*TYR*, *TRP‐1*, and *TRP‐2*) following 48 h PPV exposure. In the case of 10 μg/mL PPV treatment, all mRNA levels were significantly decreased. The lowest values were obtained at 25 μg/mL of PPV, which reduced *TYR*, *TRP‐1*, and *TRP‐2* transcripts by 14.9%, 11.3%, and 31.7%, respectively, in comparison with the control (Figure 2C–E). This coordinated suppression of melanogenic markers further supported the multi‐target inhibitory effect of PPV on the melanin synthesis pathway.

### 3.3 Effects of PPV on Melanogenesis‐Related Signaling Pathways

**FIGURE 3:**
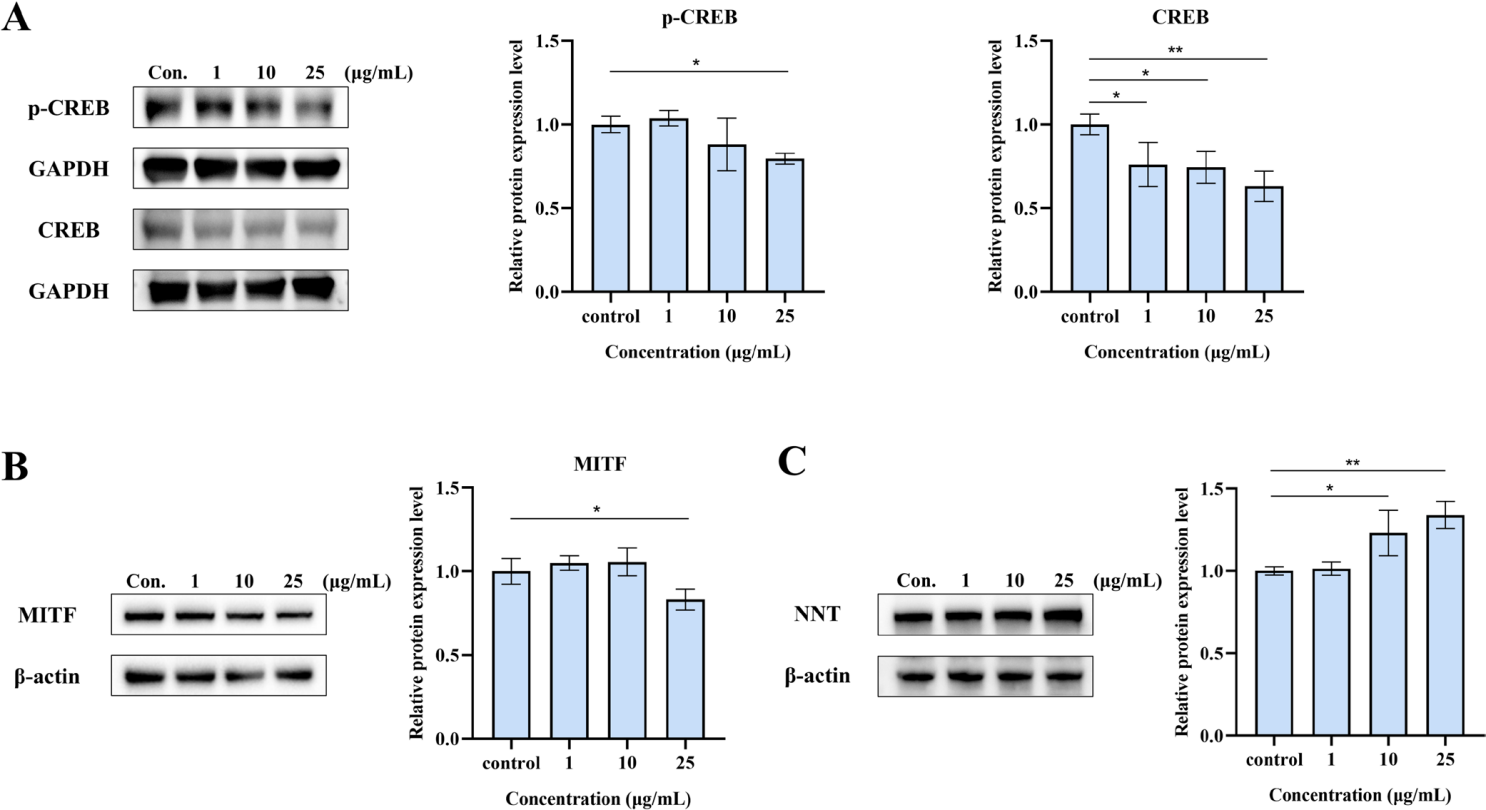
Effects of PPV on melanogenic proteins in B16F10 cells. (A) The relative protein expression levels of total CREB and p‐CREB were measured after PPV treatment for 2 h. After 48 h treatment, the protein expression levels of (B) MITF and (C) NNT were measured in PPV‐treated B16F10 cells. Data were presented as the mean ± SD of three independent experiments. The statistical analysis was performed using one‐way ANOVA followed by Dunnet's test. *p < 0.05 and **p < 0.01 versus control groups. CREB, cAMP‐response element binding protein; MITF, microphthalmia‐associated transcription factor; NNT, nicotinamide nucleotide transhydrogenase; p‐CREB, phosphorylated cAMP‐response element binding protein.

The CREB‐MITF pathway is a classic melanogenesis signaling pathway that regulates the expression of downstream melanin synthesis‐related enzymes, including TYR, TRP‐1, and TRP‐2 [23]. Western blot analysis revealed that treatment with 25 μg/mL PPV significantly inhibited the protein expression of both p‐CREB and total CREB, with inhibition rates of 20.4% and 36.9%, respectively (Figure 3A). Furthermore, MITF expression, a downstream factor of p‐CREB, was downregulated by 16.8% at 25 μg/mL PPV (Figure 3B). Recent research findings indicated that melanogenesis relied heavily on NNT activation. This activation alters cellular redox balance, impacting tyrosinase degradation and, subsequently, melanosome maturation, ultimately affecting eumelanin levels and pigmentation [11]. To further investigate the whitening mechanism of PPV, we examined its effect on NNT protein levels. Western blot analysis confirmed that PPV upregulated NNT protein expression in a dose‐dependent manner, with a significant 34.0% increase at 25 μg/mL (Figure 3C). Subsequently, we further analyzed the activation effect of individual components (PMBE, PSBE, and VC‐IP) on *NNT* gene expression levels (Table 2). Compared with the control treatment, PMBE significantly upregulated the transcription level of *NNT* by 1.26‐fold and 1.55‐fold at 1 and 10 μg/mL, respectively. Meanwhile, PSBE regulated the transcription level of *NNT*, with increases of 103% at 1 μg/mL, 72% at 10 μg/mL, and 38% at 25 μg/mL, respectively. However, VC‐IP only induced significant *NNT* upregulation at a concentration of 25 μg/mL (1.28 ± 0.09). A dose‐dependent increase in *NNT* expression was observed in PPV‐treated cells, with a 3.41‐fold upregulation of *NNT* transcription at 25 μg/mL of PPV. Synergistic interaction analysis using Jin's formula revealed significant cooperative effects at 10 μg/mL (*Q* = 1.33) and 25 μg/mL (*Q* = 1.51), exceeding the synergism threshold (*Q* ≥ 1.15). These results indicated that PPV inhibited melanogenesis through multi‐pathway regulation, significantly suppressing the CREB‐MITF pathway while synergistically activating the NNT pathway.

**TABLE 2:** Effect of PMBE, PSBE, VC‐IP, and PPV on *NNT* gene expression.

Groups	1 μg/mL	10 μg/mL	25 μg/mL
PMBE	1.26 ± 0.05*	1.55 ± 0.09***	0.52 ± 0.04
PSBE	2.03 ± 0.19***	1.72 ± 0.29***	1.38 ± 0.34***
VC‐IP	1.00 ± 0.08	0.85 ± 0.09	1.28 ± 0.09*
PPV	1.28 ± 0.20*	2.46 ± 0.19***	3.41 ± 0.15***
*Q* value	0.74	1.33	1.51

*Note:* Changes in *NNT* gene expression were normalized to the control group. PMBE, *Pterocarpus marsupium* bark extract; PSBE, *Pinus strobus* bark extract; VC‐IP, ascorbyl tetraisopalmitate; PPV, a mixture of PMBE, PSBE, and VC‐IP. *p < 0.05 and ***p < 0.001 versus control groups.

### 3.4 Inhibition of NNT Attenuated the Reduction in Oxidative Stress Levels Induced by PPV

**FIGURE 4:**
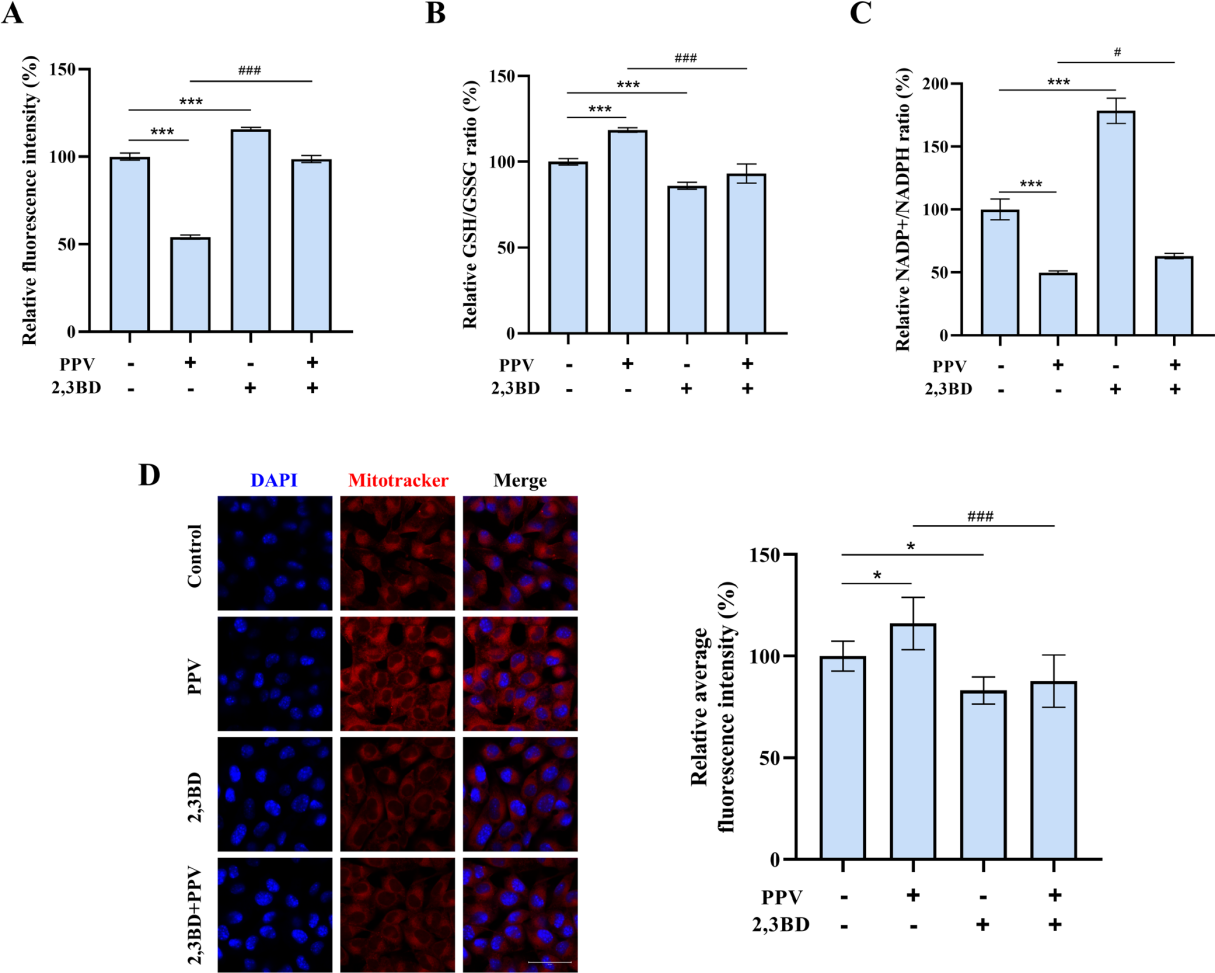
Inhibition of NNT reversed the reduction in oxidative stress levels induced by PPV. The relative ratios of intracellular (A) ROS, (B) GSH/GSSG, and (C) NADP^+^/NADPH were measured. (D) Mitochondrial oxidative stress level was observed using confocal microscopy. Red indicates staining with Mito‐Tracker Red CMXRos, and blue represents staining with DAPI. The scale bar represents 50 μm. The relative average fluorescence intensity of Mito‐Tracker Red in B16F10 cells was quantified using ImageJ software and normalized to the control group. Data were presented as the mean ± SD of at least three independent experiments. The statistical analysis was performed using one‐way ANOVA followed by LSD's post hoc test. *p < 0.05 and ***p < 0.001 versus the first bar. ^#^ *p* < 0.05 and ^###^ *p* < 0.001 versus the second bar. 2,3BD, 2,3‐butanedione; PPV, a combination of PMBE, PSBE, and VC‐IP.

Building on the identified NNT activation capacity, we explored its functional role in the anti‐melanogenic effects of PPV through targeted NNT inhibition with 2,3BD. Given the established role of NNT in GSH regeneration and redox homeostasis [11], we systematically assessed oxidative stress markers (ROS levels, GSH/GSSG ratio, NADP^+^/NADPH ratio) and mitochondrial density in B16F10 cells co‐treated with PPV (10 μg/mL) and 2,3BD (5 μM) for 48 h. PPV treatment significantly reduced intracellular ROS accumulation by 46.01%, an effect that was reversed by co‐treatment with 2,3BD (Figure 4A). The redox‐modulating capacity of PPV was further demonstrated by its ability to elevate the GSH/GSSG ratio by 18.48% and reduce the NADP^+^/NADPH ratio by 50.30%, both effects being counteracted upon NNT inhibition (Figure 4B,C). Mitochondria are a major source of ROS production within cells [24]. We used MitoTracker Red, a dye highly sensitive to changes in mitochondrial membrane potential, to reflect the level of oxidative stress in mitochondria. As shown in Figure 4D, PPV treatment resulted in a 10.82% increase in fluorescence intensity compared to the control group, indicating an enhanced mitochondrial membrane potential and a decreased mitochondrial oxidative stress level. However, this decrease in oxidative stress level was significantly reversed after 2,3BD treatment. These results suggested that PPV treatment enabled cells to effectively scavenge oxidation products that accumulated in the cytoplasm, with this effect being mediated through the upregulation of NNT.

### 3.5 Inhibition of NNT Reversed the Suppression in Pigmentation and Melanosome Maturation Induced by PPV

**FIGURE 5:**
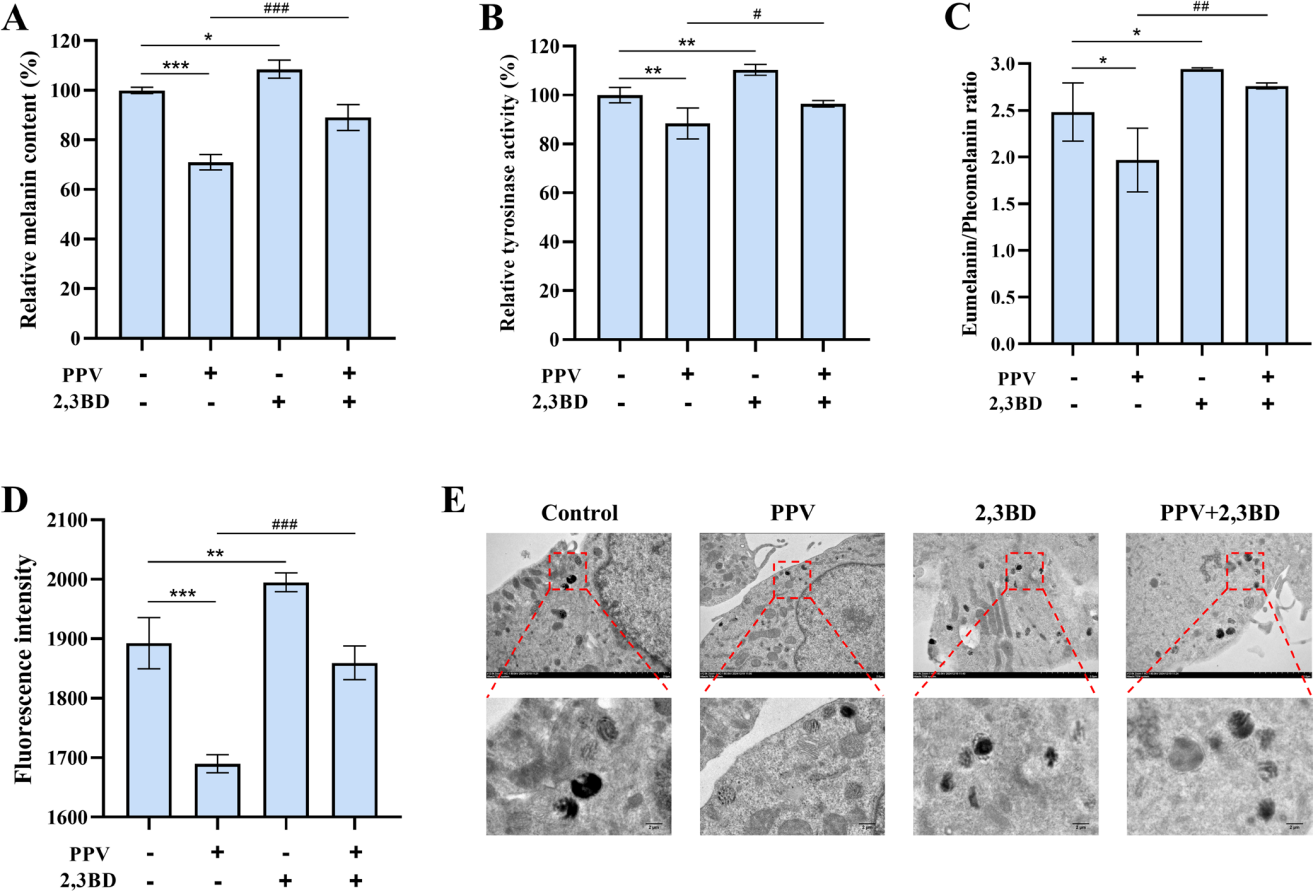
Inhibition of NNT reversed the suppression of pigmentation and melanosome maturation induced by PPV. (A) The relative melanin content and (B) relative tyrosinase activity were measured in B16F10 cells. (C) The eumelanin/pheomelanin ratio was determined using ELISA kits. (D) Cells were loaded with BCECF AM, and fluorescence intensity was measured. (E) Melanosome maturation was observed by transmission electron microscopy. The top images show observations at ×12 000 magnification, with the bottom images representing the areas within the dashed boxes from the top images. The scale bars represent 2.0 μm. Data were presented as the mean ± SD of at least three independent experiments. The statistical analysis was performed using one‐way ANOVA followed by LSD's post hoc test. *p < 0.05, **p < 0.01, and ***p < 0.001 versus the first bar. ^#^ *p* < 0.05, ^##^ *p* < 0.01, and ^###^ *p* < 0.001 versus the second bar. 2,3BD, 2,3‐butanedione; PPV, a combination of PMBE, PSBE, and VC‐IP.

To investigate whether PPV regulates pigmentation via NNT, we assessed melanin content and tyrosinase activity following treatment with 2,3BD (5 μM) and PPV (10 μg/mL). Co‐treatment with 2,3BD significantly attenuated the anti‐melanogenic effects of PPV, leading to an 18.04% increase in melanin content and a 7.94% restoration of tyrosinase activity compared to PPV treatment alone (Figure 5A,B). Given that PPV elevated GSH levels, we further examined the eumelanin/pheomelanin ratio, which decreased as GSH content increased. PPV treatment reduced this ratio by 0.51, consistent with its effect on GSH levels, while co‐treatment with 2,3BD blocked this reduction (Figure 5C). These results emphasized the crucial role of NNT in the regulation of the transformation from eumelanin to pheomelanin mediated by PPV. Melanosome acidification represents a determinant of melanosome maturation [8]. Intracellular pH measurements, performed using BCECF AM fluorescence, demonstrated that PPV induced acidification in B16F10 cells, an effect reversed by NNT inhibition (Figure 5D). Notably, electron microscopy analysis showed that PPV treatment resulted in a significant increase in the number of melanosomes from stage I to II in B16F10, suggesting that PPV inhibited melanosome development (Figure 5E). In contrast, inhibition of NNT led to a significant increase in late‐stage melanosomes (stages III–IV). Collectively, these findings suggested that PPV suppressed pigmentation and melanosome maturation through modulation of NNT.

### 3.6 Reduction of Pigmentation in Human Ex Vivo Skin Model by PPV

**FIGURE 6:**
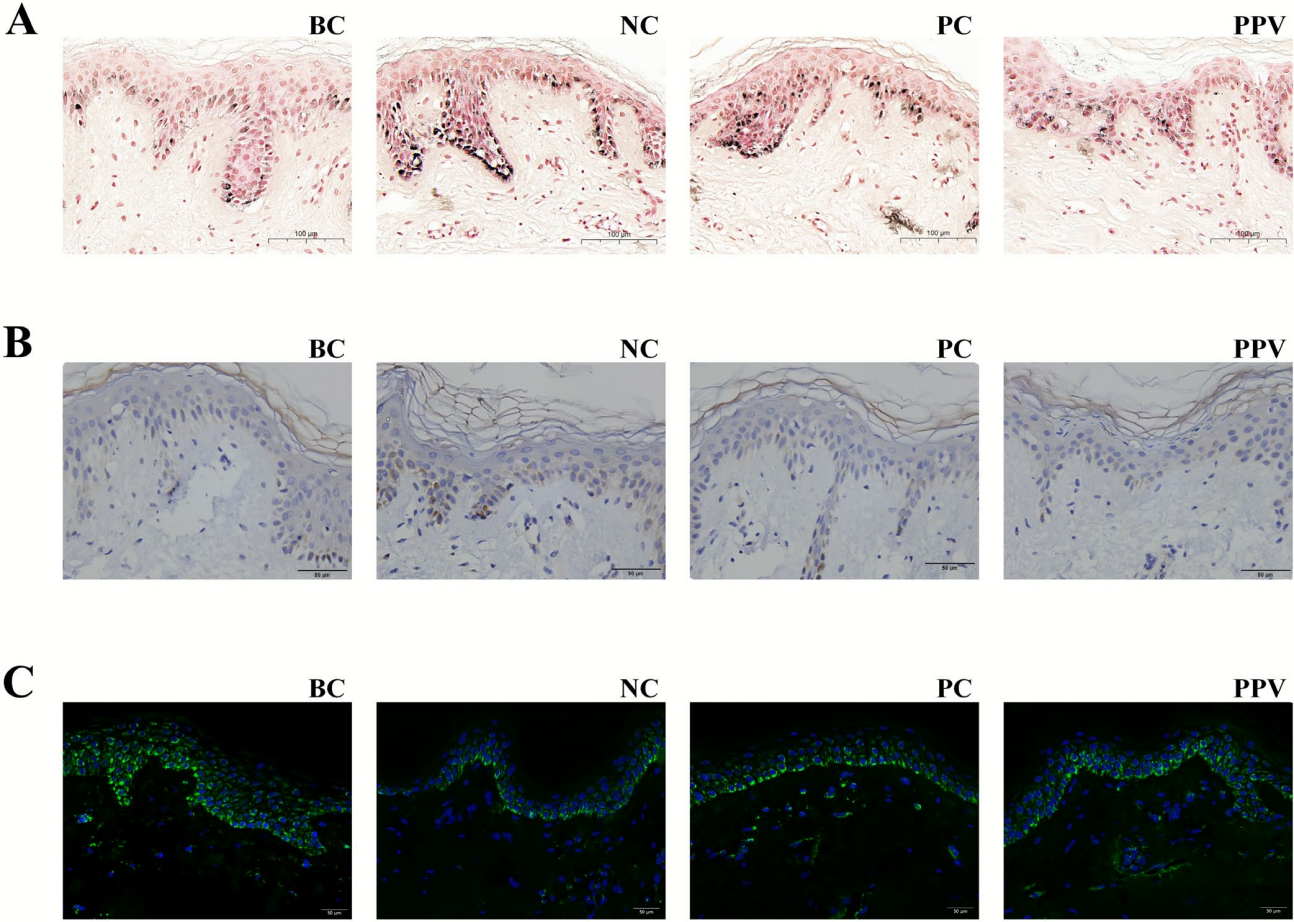
Reduction of pigmentation and activation of NNT expression in human ex vivo skin model by PPV. (A) Fontana‐Masson staining was performed on ex vivo skin samples. Scale bars represent 100 μm. (B) Tyrosinase immunostaining of ex vivo skin. Scale bars represent 50 μm. (C) Immunofluorescence images showing NNT expression in the ex vivo skin model (green: NNT; blue: Nuclei). Scale bars represent 50 μm. BC, blank control; NC, negative control; PC, positive control; PPV, a combination of PMBE, PSBE, and VC‐IP.

To verify whether PPV reduces epidermal melanin accumulation in a human ex vivo skin model, we performed Fontana‐Masson staining. The results indicated that compared to the young ex vivo skin (BC), the aged ex vivo skin (NC) exhibited substantial, dense, and dark melanin deposition, demonstrating a significant age‐related increase in pigmentation (Figure 6A). Following treatment with PPV (10 μg/mL), the intensity and density of melanin deposition were markedly reduced compared to the NC group, with a visible lightening of color, and the effect was superior to that of the positive control (PC) kojic acid (42.67 μg/mL). Immunohistochemical staining for tyrosinase showed brown‐yellow positive signals. The NC group displayed extensive brown‐yellow staining, whereas the PPV‐treated group exhibited a significant reduction in these positive signals, indicating that PPV effectively downregulated tyrosinase expression (Figure 6B). Subsequently, we performed immunofluorescence staining for NNT, which displayed green fluorescence. The green fluorescence in the NC group was significantly lower than that in the BC group (Figure 6C). After PPV treatment, the green fluorescence intensity markedly increased, demonstrating that PPV treatment effectively reversed the reduced NNT expression in aged skin and strongly upregulated its protein level. These findings indicated that PPV not only attenuates age‐related melanin accumulation but also enhances NNT protein expression in human skin.

## 4 Discussion

Excessive production and accumulation of melanin can lead to various skin disorders, such as melasma, freckles, age spots, and other forms of hyperpigmentation [25]. Recent studies have identified NNT as a key regulatory mechanism involved in skin pigmentation [11]. Notably, the combination of nicotinamide, vitamin C, and polydeoxyribonucleotide has been shown to mitigate melanogenesis by modulating NNT [26]. While the roles of PMBE, PSBE, and VC‐IP in inhibiting melanogenesis were well established, their potential involvement in the NNT pathway remains unexplored. Additionally, the use of a composite formulation strategy not only reduces the concentration of individual components but also leverages the synergistic effects of the combined ingredients [27]. In the present study, we demonstrated that PPV, a composite formulation of PMBE, PSBE, and VC‐IP, effectively reduced melanogenesis mainly by modulating NNT.

PMBE, PSBE, and VC‐IP have been widely used in whitening cosmetics. Here, our study revealed that co‐administration of the three agents exhibited potential in reducing melanin synthesis, primarily through suppression of tyrosinase enzymatic function and transcriptional downregulation of melanogenic markers *TYR*, *TRP‐1*, and *TRP‐2*. This indicated that PPV suppressed melanin production by reducing the activity of key enzymes involved in melanin synthesis. Notably, the pronounced downregulation of *TRP‐2* suggested that PPV may preferentially target the later stages of melanogenesis, where *TRP‐2* plays a critical role in stabilizing tyrosinase and modulating melanosome structure [28]. PPV treatment resulted in a significant decrease in the number of melanosomes from stage III to IV in B16F10 cells, providing validation of this regulatory mechanism. It was evident that PPV exhibited anti‐melanogenic activity within the concentration range of 10–25 μg/mL. This concentration was significantly lower than the commonly used concentrations of active ingredients in cosmetics and topical pharmaceuticals. For instance, niacinamide, a widely used whitening ingredient in cosmetics, was typically employed at concentrations ranging from 2% to 5% [29]. As a positive control in whitening research, arbutin was considered safe at a 2% concentration in cosmetic formulations [30]. Consequently, incorporating 25 μg/mL (0.0025%) PPV into creams, gels, or serums was feasible.

Additionally, we better characterized the depigmenting mechanism and the synergistic effects of this combination. NNT is a melanogenic regulator that operates independently of the canonical MITF‐mediated melanogenesis pathway [11]. Our study demonstrated that PPV simultaneously inhibited the CREB‐MITF signaling pathway while activating the NNT pathway. However, since NNT activation exhibited a more pronounced effect than MITF inhibition at 25 μg/mL PPV, we proposed that the NNT signaling pathway played a predominant role in mediating the whitening effects of PPV. While NNT activation was primary, the inhibition of the CREB‐MITF pathway likely contributed to a synergistic effect. Mechanistically, NNT activation concurrently targeted post‐translational degradation of melanogenic enzymes and melanosome maturation, while MITF inhibition suppressed their transcriptional expression. This multi‐targeted attack on both the synthesis and the processing of melanin explained the superior efficacy of the PPV combination. We further investigated the effects of individual components and their combinations on *NNT* gene expression levels. The combined formulation, PPV, showed a dose‐dependent upregulation of *NNT* expression, highlighting its enhanced efficacy compared to individual components. PMBE, PSBE, and VC‐IP could modulate NNT expression at different levels, which could explain the observed synergistic effects of this combination. However, the observed upregulation of NNT expression by PPV was significantly more pronounced at the mRNA level than at the protein level, suggesting potential regulation at post‐transcriptional, translational, or post‐translational stages [31]. Future studies directly profiling ribosome occupancy on the NNT mRNA and measuring NNT protein turnover rates will be crucial to distinguish between these mechanisms and clarify the underlying regulatory pathways. Meanwhile, multiple studies identified NNT as a direct transcriptional target of Nuclear factor erythroid 2‐related factor 2 (Nrf2) [32]. Nrf2 is a master regulator of the antioxidant response. Upon activation by oxidative stress, Nrf2 translocates to the nucleus and binds to Antioxidant Response Elements (AREs) in target gene promoters. Some evidence suggested Forkhead box O3 (FoxO3), another transcription factor sensitive to cellular redox status, may influence NNT expression [33]. While less extensively characterized for NNT than Nrf2, FoxO3 activation can promote the expression of genes involved in oxidative stress defense. Notably, NNT expression can also be modulated post‐transcriptionally. Long non‐coding RNAs and microRNAs can influence NNT mRNA stability or translation [34, 35]. We therefore speculate that PPV's antioxidant effects might modulate these transcription factors or non‐coding RNAs, thereby enhancing the expression of NNT. While our current study focused on establishing the functional role of PPV‐induced NNT upregulation in inhibiting melanogenesis, the precise molecular mediators initiating this induction require further investigation.

NNT plays a critical role in maintaining cellular redox homeostasis. Genetic silencing of NNT in human adrenocortical cells disrupted redox potential, concomitant with elevated mitochondrial ROS accumulation and activation of apoptotic pathways [36]. Excessive ROS production is known to stimulate melanin synthesis by activating tyrosinase and other melanogenic pathways [37]. Existing research indicated that PMBE suppressed ROS generation and modulated the proopiomelanocortin (POMC)/α‐melanocyte‐stimulating hormone (α‐MSH) signaling pathway in HaCaT keratinocytes [12]. Cellular ROS primarily originate from NADPH oxidases (NOX) alongside the mitochondrial electron transport chain [38]. NOX activation leads to NADPH donating reductive potential to glutathione and thioredoxins. These molecules, in turn, facilitate ROS neutralization by glutaredoxins, peroxiredoxins, and glutathione peroxidases [39]. In this process, GSH can undergo oxidation to form GSSG, and it can be restored through the activity of glutathione reductase in conjunction with the NADPH system [40]. NNT acts as a catalyst in this reaction, helping to regulate the balance of NADH^+^ and NADPH levels [41]. In this study, PPV treatment significantly reduced intracellular ROS levels, increased the GSH/GSSG ratio, and decreased the NADP^+^/NADPH ratio in B16F10 cells, while these changes were reversed by the NNT inhibitor (2,3BD). According to the study, PPV triggered the intracellular antioxidant defense system in B16F10 cells, and the activation of NNT plays a significant role in its whitening effects. NNT may modulate pigmentation by influencing the regeneration of GSH, thus impacting the pheomelanin‐to‐eumelanin ratio [11]. PPV treatment reduced the eumelanin/pheomelanin ratio, an effect that was blocked by 2,3BD. By enhancing GSH levels, PPV shifted melanin synthesis toward the production of pheomelanin, thereby contributing to its skin‐lightening effects. The reversal of this effect by 2,3BD underscored the critical role of NNT in maintaining GSH levels and regulating melanin biosynthesis. Even though the B16F10 cell line exhibits significant differences from normal human melanocytes in redox regulation and other aspects, it remains a good model for studying the effects of compounds on melanogenesis [42]. B16F10 cells, due to their rapid proliferation, stable passage, and partially conserved melanogenesis pathway with human cells, have been widely used in the analysis of whitening mechanisms, skin protection, and melanoma treatment [43]. However, clinical translation requires further validation using human cell models and in vivo experiments. Due to the absence of physiological skin barriers and metabolic processes in monolayer cell cultures, the safety and efficacy of PPV observed in vitro cannot be directly extrapolated to normal human skin. Further studies, including preclinical assessment of local tolerance and dose‐ranging trials in healthy volunteers, are required to establish its topical applicability.

Ultimately, we assessed the impact of PPV on pigmentation using an ex vivo skin model. The findings demonstrated a significant reduction in epidermal basal pigmentation in skin tissues treated with PPV. However, we measured the total melanin within the epidermal tissue, so the proportion of melanin derived from keratinocytes versus melanocytes remained unclear. Furthermore, we did not investigate the effect of PPV on melanosome transfer. It would be necessary to use a co‐culture model to evaluate the impact of PPV on melanin transfer from melanocytes into keratinocytes. In addition, the increased expression of NNT induced by PPV correlated with decreased melanin levels in aged ex vivo skin, indicating that PPV may utilize this mechanism to diminish age‐related melanin accumulation. However, the ex vivo skin experiments were performed using tissues from a single young and a single aged donor. Consequently, we cannot assess inter‐individual variability, and the generalizability of these results requires future validation with a larger sample size. However, the trends observed in the ex vivo model were highly consistent with the data obtained from experiments in B16F10 cells. This consistency strongly suggested that the anti‐melanogenic effects of PPV were a robust phenomenon. Future work will focus on recruiting a larger cohort of donor samples to further corroborate the generalizability of these findings. Additional studies are also needed to assess its long‐term safety, efficacy, and clinical applicability in vivo.

In conclusion, our findings clearly demonstrated that PPV, a novel formulation combining PMBE, PSBE, and VC‐IP, effectively suppressed melanogenesis through the activation of NNT and suppression of MITF. These data collectively highlighted the ability of PPV to improve human skin as well as melanocyte pigmentation. Therefore, PPV holds promise as a therapeutic candidate for hyperpigmentation disorders, given its ability to target multiple pathways involved in melanogenesis.

## Author Contributions

**Kejie Peng:** writing – original draft, investigation. **Jian Mu:** supervision, validation. **Yumeng Li:** writing – review and editing. **Zhangtie Wang:** writing – review and editing, supervision, validation. **Yansong Peng:** writing – review and editing, supervision, validation. **Tian Zhao:** writing – review and editing. **Yixuan Wang:** writing – review and editing, supervision, validation. **Jing Wang:** supervision, validation. **Baiyi Lu:** supervision, validation.

## Funding

This work was supported by ‘Pioneer’ and ‘Leading Goose’ R&D Program of Zhejiang Province (No. 2025C01100) and Proya Cosmetics Co. Ltd.

## Ethics Statement

The study was approved by the local Ethics Committee (approval No. GDLL2024002 and No. GDLL2024003).

## Conflicts of Interest

The authors declare no conflicts of interest.

## Data Availability

The data that support the findings of this study are available from the corresponding author upon reasonable request.

## References

[1] W. Choi , Y. Miyamura , R. Wolber , et al., “Regulation of Human Skin Pigmentation In Situ by Repetitive UV Exposure: Molecular Characterization of Responses to UVA and/or UVB,” Journal of Investigative Dermatology 130, no. 6 (2010): 1685–1696, 10.1038/jid.2010.5. 20147966 PMC3478754

[2] H. M. Wang , H. J. Lai , A. G. Wu , et al., “Melanogenic Effects of 5‐Demethylnobiletin on Mouse Model of Chemical‐Induced Vitiligo,” Journal of Functional Foods 112 (2024): 105962, 10.1016/j.jff.2023.105962.

[3] M. Sugumaran and H. Barek , “Critical Analysis of the Melanogenic Pathway in Insects and Higher Animals,” International Journal of Molecular Sciences 17, no. 10 (2016): 1753, 10.3390/ijms17101753. 27775611 PMC5085778

[4] T. Pillaiyar , M. Manickam , and S. H. Jung , “Recent Development of Signaling Pathways Inhibitors of Melanogenesis,” Cellular Signalling 40 (2017): 99–115, 10.1016/j.cellsig.2017.09.004. 28911859

[5] X. Wang , L. Kinziabulatova , M. Bortoli , et al., “Indole‐5,6‐Quinones Display Hallmark Properties of Eumelanin,” Nature Chemistry 15, no. 6 (2023): 787–793, 10.1038/s41557-023-01175-4. 37037912

[6] J. R. Jara , P. Aroca , F. Solano , J. H. Martinez , and J. A. Lozano , “The Role of Sulfhydryl Compounds in Mammalian Melanogenesis: The Effect of Cysteine and Glutathione Upon Tyrosinase and the Intermediates of the Pathway,” Biochimica et Biophysica Acta‐General Subjects 967, no. 2 (1988): 296–303, 10.1016/0304-4165(88)90023-2. 2903772

[7] H. M. Wang , L. Q. Qu , J. P. L. Ng , et al., “Natural Citrus Flavanone 5‐Demethylnobiletin Stimulates Melanogenesis Through the Activation of cAMP/CREB Pathway in B16F10 Cells,” Phytomedicine 98 (2022): 153941, 10.1016/j.phymed.2022.153941. 35114451

[8] K. Wakamatsu , J. H. Zippin , and S. Ito , “Chemical and Biochemical Control of Skin Pigmentation With Special Emphasis on Mixed Melanogenesis,” Pigment Cell & Melanoma Research 34, no. 4 (2021): 730–747, 10.1111/pcmr.12970. 33751833 PMC8861806

[9] M. P. Murphy , “Redox Modulation by Reversal of the Mitochondrial Nicotinamide Nucleotide Transhydrogenase,” Cell Metabolism 22, no. 3 (2015): 363–365, 10.1016/j.cmet.2015.08.012. 26331603

[10] F. L. Sheeran , J. Rydström , M. I. Shakhparonov , N. B. Pestov , and S. Pepe , “Diminished NADPH Transhydrogenase Activity and Mitochondrial Redox Regulation in Human Failing Myocardium,” Biochimica et Biophysica Acta‐Bioenergetics 1797, no. 6 (2010): 1138–1148, 10.1016/j.bbabio.2010.04.002. 20388492

[11] J. Allouche , I. Rachmin , K. Adhikari , et al., “NNT Mediates Redox‐Dependent Pigmentation via a UVB‐ and MITF‐Independent Mechanism,” Cell 184, no. 16 (2021): 4268–4283.e20, 10.1016/j.cell.2021.06.022. 34233163 PMC8349839

[12] Y. C. Hseu , Y. V. Gowrisankar , L. W. Wang , et al., “The In Vitro and In Vivo Depigmenting Activity of Pterostilbene Through Induction of Autophagy in Melanocytes and Inhibition of UVA‐Irradiated α‐MSH in Keratinocytes via Nrf2‐Mediated Antioxidant Pathways,” Redox Biology 44 (2021), 10.1016/j.redox.2021.102007. PMC8167190 34049220

[13] T. Miura , M. Chiba , K. Kasai , et al., “Apple Procyanidins Induce Tumor Cell Apoptosis Through Mitochondrial Pathway Activation of Caspase‐3,” Carcinogenesis 29, no. 3 (2008): 585–593, 10.1093/carcin/bgm198. 17827407

[14] J. Legault , K. Girard‐Lalancette , D. Dufour , and A. Pichette , “Antioxidant Potential of Bark Extracts From Boreal Forest Conifers,” Antioxidants 2, no. 3 (2013): 77–89, 10.3390/antiox2030077. 26784337 PMC4665433

[15] M. Yokota and S. Yahagi , “Evaluation of the Anti‐Wrinkle Effect of a Lipophilic Pro‐Vitamin C Derivative, Tetra‐Isopalmitoyl Ascorbic Acid,” Journal of Cosmetic Dermatology 21, no. 8 (2022): 3503–3514, 10.1111/jocd.14604. 34910367

[16] Y. Ochiai , S. Kaburagi , K. Obayashi , et al., “A New Lipophilic Pro‐Vitamin C, Tetra‐Isopalmitoyl Ascorbic Acid (VC‐IP), prevents UV‐Induced Skin Pigmentation Through Its Anti‐Oxidative Properties,” Journal of Dermatological Science 44, no. 1 (2006): 37–44, 10.1016/j.jdermsci.2006.07.001. 16935471

[17] J. H. Kim , K. S. Han , E. S. Lee , et al., “Inhibitory Effects of Phenylpropionamides From the Hemp Seed Husk on Tyrosinase,” International Journal of Biological Macromolecules 282 (2024): 136939, 10.1016/j.ijbiomac.2024.136939. 39490470

[18] J. Wu , X. Li , H. Fang , et al., “Investigation of Synergistic Mechanism and Identification of Interaction Site of Aldose Reductase With the Combination of Gigantol and Syringic Acid for Prevention of Diabetic Cataract,” BMC Complementary and Alternative Medicine 16, no. 1 (2016): 286, 10.1186/s12906-016-1251-5. 27520089 PMC4983052

[19] Q. Chen , Q. Zhang , A. T. Amrouche , W. Huang , and B. Lu , “Asperuloside, the Bioactive Compound in the Edible *Eucommia ulmoides* Male Flower, Delays Muscle Aging by Daf‐16 Mediated Improvement in Mitochondrial Dysfunction,” Food & Function 14, no. 12 (2023): 5562–5575, 10.1039/D3FO01024D. 37212195

[20] Y. Dan , L. Chen , S. Jin , et al., “Photobiomodulation Using 830 Nm Lighting‐Emitting Diode Inhibits Melanogenesis via FOXO3a in Human Melanocyte,” Pigment Cell & Melanoma Research 37, no. 5 (2024): 681–692, 10.1111/pcmr.13193. 39169669

[21] F. Jiang , Y. Wu , Z. Liu , M. Hong , and Y. Huang , “Synergy of GHK‐Cu and Hyaluronic Acid on Collagen IV Upregulation via Fibroblast and Ex‐Vivo Skin Tests,” Journal of Cosmetic Dermatology 22, no. 9 (2023): 2598–2604, 10.1111/jocd.15763. 37062921

[22] Y. Cao , J. Lv , Y. Tan , et al., “Tribuloside Acts on the PDE/cAMP/PKA Pathway to Enhance Melanogenesis, Melanocyte Dendricity and Melanosome Transport,” Journal of Ethnopharmacology 323 (2024): 117673, 10.1016/j.jep.2023.117673. 38158096

[23] J. Li , S. Jiang , C. Huang , and X. Yang , “Atraric Acid Ameliorates Hyperpigmentation Through the Downregulation of the PKA/CREB/MITF Signaling Pathway,” International Journal of Molecular Sciences 23, no. 24 (2022): 15952, 10.3390/ijms232415952. 36555593 PMC9788525

[24] Y. Liu , D. Bao , H. She , et al., “Role of Hippo/ACSL4 Axis in Ferroptosis‐Induced Pericyte Loss and Vascular Dysfunction in Sepsis,” Redox Biology 78 (2024): 103353, 10.1016/j.redox.2024.103353. 39566164 PMC11617880

[25] H. C. Huang , Y. C. Chou , C. Y. Wu , and T. M. Chang , “[8]‐Gingerol Inhibits Melanogenesis in Murine Melanoma Cells Through Down‐Regulation of the MAPK and PKA Signal Pathways,” Biochemical and Biophysical Research Communications 438, no. 2 (2013): 375–381, 10.1016/j.bbrc.2013.07.079. 23892040

[26] H. J. Park , K. A. Byun , S. Oh , et al., “The Combination of Niacinamide, Vitamin C, and PDRN Mitigates Melanogenesis by Modulating Nicotinamide Nucleotide Transhydrogenase,” Molecules 27, no. 15 (2022): 4923, 10.3390/molecules27154923. 35956878 PMC9370691

[27] X. Shu , X. Dong , Y. Ma , et al., “The Whitening Efficacy of a Compound Formula Examined Using an Ultraviolet‐Induced Skin Melanization Model,” Journal of Cosmetic Dermatology 23, no. 8 (2024): 2750–2756, 10.1111/jocd.16332. 38664985

[28] S. A. N. D'Mello , G. J. Finlay , B. C. Baguley , and M. E. Askarian‐Amiri , “Signaling Pathways in Melanogenesis,” International Journal of Molecular Sciences 17, no. 7 (2016): 1144, 10.3390/ijms17071144. 27428965 PMC4964517

[29] S. Bhattaccharjee , M. Beck‐Broichsitter , and A. K. Banga , “In Situ Gel Formation in Microporated Skin for Enhanced Topical Delivery of Niacinamide,” Pharmaceutics 12, no. 5 (2020): 472, 10.3390/pharmaceutics12050472. 32455797 PMC7284857

[30] J. Couto , A. Figueirinha , M. T. Batista , et al., “ *Fragaria vesca* L. Extract: A Promising Cosmetic Ingredient With Antioxidant Properties,” Antioxidants 9, no. 2 (2020): 154, 10.3390/antiox9020154. 32074975 PMC7070388

[31] X. Tong and J. C. Pelling , “Enhancement of p53 Expression in Keratinocytes by the Bioflavonoid Apigenin Is Associated With RNA‐Binding Protein HuR,” Molecular Carcinogenesis 48, no. 2 (2009): 118–129, 10.1002/mc.20460. 18680106 PMC2631086

[32] S. Heidarizadi , Z. Rashidi , C. Jalili , et al., “Melatonin Protects Mouse Type A Spermatogonial Stem Cells Against Oxidative Stress via the Mitochondrial Thioredoxin System,” Cell Journal 25, no. 11 (2023): 741–752, 10.22074/cellj.2023.2003766.1316. 38071406 PMC10711295

[33] P. C. Bradshaw , W. A. Seeds , A. C. Miller , V. R. Mahajan , and W. M. Curtis , “COVID‐19: Proposing a Ketone‐Based Metabolic Therapy as a Treatment to Blunt the Cytokine Storm,” Oxidative Medicine and Cellular Longevity 2020 (2020): 6401341, 10.1155/2020/6401341. 33014275 PMC7519203

[34] Z. Xiong , W. Xiong , W. Xiao , et al., “NNT‐Induced Tumor Cell “Slimming” Reverses the Pro‐Carcinogenesis Effect of HIF2a in Tumors,” Clinical and Translational Medicine 11, no. 1 (2021): e264, 10.1002/ctm2.264. 33463050 PMC7803359

[35] X. Pan , Q. Wang , Y. Yu , et al., “Antisense lncRNA NNT‐AS1 Promoted Esophageal Squamous Cell Carcinoma Progression by Regulating Its Sense Gene NNT Expression,” Cell Death Discov 8, no. 1 (2022): 424, 10.1038/s41420-022-01216-w. 36270987 PMC9586939

[36] G. McCambridge , M. Agrawal , A. Keady , et al., “Saturated Fatty Acid Activates T Cell Inflammation Through a Nicotinamide Nucleotide Transhydrogenase (NNT)‐Dependent Mechanism,” Biomolecules 9, no. 2 (2019): 79, 10.3390/biom9020079. 30823587 PMC6406569

[37] R. Campiche , S. J. Curpen , V. Lutchmanen‐Kolanthan , et al., “Pigmentation Effects of Blue Light Irradiation on Skin and How to Protect Against Them,” International Journal of Cosmetic Science 42, no. 4 (2020): 399–406, 10.1111/ics.12637. 32478879 PMC7496068

[38] M. Skonieczna , T. Hejmo , A. Poterala‐Hejmo , A. Cieslar‐Pobuda , and R. J. Buldak , “NADPH Oxidases: Insights Into Selected Functions and Mechanisms of Action in Cancer and Stem Cells,” Oxidative Medicine and Cellular Longevity 2017, no. 1 (2017): 9420539, 10.1155/2017/9420539. 28626501 PMC5463201

[39] P. J. Fernandez‐Marcos and S. Nóbrega‐Pereira , “NADPH: New Oxygen for the ROS Theory of Aging,” Oncotarget 7, no. 32 (2016): 50814–50815, 10.18632/oncotarget.10744. 27449104 PMC5239434

[40] S. Panday , R. Talreja , and M. Kavdia , “The Role of Glutathione and Glutathione Peroxidase in Regulating Cellular Level of Reactive Oxygen and Nitrogen Species,” Microvascular Research 131 (2020): 104010, 10.1016/j.mvr.2020.104010. 32335268

[41] H. Y. Ho , Y. T. Lin , G. Lin , P. R. Wu , and M. L. Cheng , “Nicotinamide Nucleotide Transhydrogenase (NNT) Deficiency Dysregulates Mitochondrial Retrograde Signaling and Impedes Proliferation,” Redox Biology 12 (2017): 916–928, 10.1016/j.redox.2017.04.035. 28478381 PMC5426036

[42] Y. C. Chung , J. H. Ko , H. K. Kang , et al., “Antimelanogenic Effects of Polygonum Tinctorium Flower Extract From Traditional Jeju Fermentation via Upregulation of Extracellular Signal‐Regulated Kinase and Protein Kinase B Activation,” International Journal of Molecular Sciences 19, no. 10 (2018): 2895, 10.3390/ijms19102895. 30249988 PMC6213794

[43] M. S. Guo , Q. Wu , Y. Xia , et al., “Cholinergic Signaling Mediated by Muscarinic Receptors Triggers the Ultraviolet‐Induced Release of Melanosome in Cultured Melanoma Cells,” Pigment Cell & Melanoma Research 38, no. 1 (2025): e13201, 10.1111/pcmr.13201. 39344704 PMC11681844

